# Cognitive frailty as a predictor of adverse outcomes among older adults: A systematic review and meta‐analysis

**DOI:** 10.1002/brb3.1926

**Published:** 2020-11-06

**Authors:** ZiHan Bu, AnLe Huang, MengTing Xue, QingYun Li, YaMei Bai, GuiHua Xu

**Affiliations:** ^1^ Nursing School Nanjing University of Chinese Medicine Nanjing China

**Keywords:** adverse outcomes, Alzheimer's disease, cognitive decline, cognitive frailty, meta‐analysis, older adults

## Abstract

**Objective:**

A systematic review and meta‐analysis basing on the prospective cohort studies were conducted to explore the risk of all‐cause mortality and dementia in cognitively frail older adults compared to robust older adults and to determine whether it was a predictor of adverse outcomes.

**Methods:**

Pubmed, Web of Science, The Cochrane Library, EMBASE, and CINAHL databases were searched to retrieve studies on adverse outcomes of cognitive frailty. Two reviewers independently screened the literature, extracted data, and assessed the risk of bias of the included studies. Stata 15.0 Software was used to perform the meta‐analysis. The all‐cause mortality and dementia were observed to be the primary outcomes, while the other data were considered as the secondary outcome.

**Results:**

A total of 14 studies were included in qualitative analysis and 12 studies were included in the meta‐analysis, with low risk of bias and moderate to good methodological quality. The results showed that cognitive frailty in older people had a higher risk of all‐cause mortality [HR = 1.93, 95%CI (1.67, 2.23), *p* < .001] and dementia [HR = 3.66, 95%CI (2.86, 4.70) as compared with robust. The subgroup analysis showed that the assessment tools were the main source of heterogeneity.

**Conclusion:**

In older adults living in communities, the cognitive frailty was found to be a significant predictor of all‐cause mortality and dementia. Nonetheless, cognitive frailty was found to be a better predictor of all‐cause mortality and dementia than just frailty.

## Graphical abstract

To systematically evaluate the effect of cognitive frailty on adverse outcomes among older adults. Cognitive frailty could increase the risk of all‐cause mortality, dementia, and other adverse outcomes in the elderly, and early screening and intervention measures should be taken.

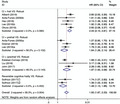

## 1 INTRODUCTION

Frailty is a state of higher vulnerability to stressors resulting from age‐associated decline in multiple physiological systems and a lower homeostatic reserve (Bartley et al., 2016; Fried et al., 2001). There are three types of frailty, namely physical frailty, cognitive frailty, and social frailty (Morley et al., 2013). According to the consensus reached by the International Institute of Nutrition and Aging Chemistry (IANA) (Kelaiditi et al., 2013) in 2013, the consensus panel proposed the identification of the “cognitive frailty” as a heterogeneous clinical manifestation characterized by the simultaneous presence of both physical frailty and cognitive impairment. The key factors defining such a condition include (a) coexistence of physical frailty and cognitive impairment (Clinical Dementia Rating [CDR] = 0.5); and (b) exclusion of concurrent Alzheimer's disease or other dementias (Kelaiditi et al., 2013), that is, cognitive impairment caused by physical factors. Existing research (Boyle et al., 2010; Canevelli et al., 2014; Solfrizzi et al., 2017) shows that physical frailty increases the risk of dementia in populations with normal cognition and accelerates cognitive decline in individuals. Current evidence has shown that cognitive functioning is the key to reversing the frailty state (Aprahamian et al., 2018). Moreover, the annual growth rate from mild cognitive impairment to dementia is relatively very high (5%~15%), and extensive neuronal loss and irreversible neuronal damage are observed at this stage (Liu et al., 2018; Panza et al., 2015). Therefore, some scholars believe that mild cognitive impairment may not be the best intervention stage at which to prevent dementia.

As mentioned above, frailty and cognitive impairment were often viewed as two independent concepts in previous studies, which helps to respectively predict adverse outcomes (Jacobs et al., 2011). But when they coexist, cumulative negative effects are often detected, significantly increasing all‐cause mortality or other adverse outcomes (St John et al., 2017), and the precursory signs need to be particularly identified that lead to adverse outcomes, and to target the specific interventions. Hence, cognitive frailty may be a physiological precursor to the degenerative nervous system diseases, which could play a key role in predicting the short‐term and the long‐term all‐cause mortality, dementia, disability, and other adverse health outcomes. Additionally, it could also offer a new target for the prevention and intervention of the pathological aging and the adverse outcomes.

This study used meta‐analysis to explore the risk of all‐cause mortality and dementia in older adults with cognitive frailty compared to robust elderly participants. Furthermore, the understanding of the relationship between frailty and geriatric cognitive disorders help healthcare professionals become more informed of cognitive frailty and the associated risks. It will also help contribute to new interventions and management of geriatric cognitive disorders.

## 2 METHODS

This systematic review was drafted in accordance with the protocol of the Preferred Reporting Items for Systematic Review and Meta‐Analyses (PRISMA) guidelines (Moher et al., 2009) for transparent reporting of the systematic reviews and meta‐analysis, registered on the PROSPERO website (CRD42020186200).

### 2.1 Search strategy

A computer‐based search of Web of Science, PubMed, Cochrane Library, CINAHL, and EMBASE was carried out from May 10, 2020. At the same time, manual retrieval of cognitive decline‐related reviews and a systematic evaluation of references was conducted to ensure the comprehensiveness of results. We used Medical Subject Headings (MeSH), free term, and word variants for two main themes, that is, “cognitive frailty” and “cohort study,” and these were combined with Boolean operator OR/AND. The search terms included “cognitive frailty” OR “cognitive decline” OR “cognitive impairment” AND (frail* OR pre‐frail* OR “frailty syndrome”) AND (“cohort study” OR “longitudinal study”) (Take PUBMED for example in Appendix S1).

### 2.2 Inclusion criteria and exclusion criteria

Inclusion criteria: (a) a prospective cohort study or population‐based longitudinal study; (b) the subjects included older people in the community aged 60 years old and above; (c) cognitive frailty needs to be defined as physical frailty and cognitive impairment simultaneously, while the internationally agreed‐upon diagnostic criteria were used to define physical frailty (e.g., Frailty Phenotype, Frail Scale, Frailty Index, or others) and the cognitive impairment (Minimum Mental State Examination, Clinical Dementia Rating, or others), and (d) reported the hazard ratio (HR), the odds ratio (OR), or risk ratio (RR) of the primary outcomes (all‐cause mortality and dementia) and other adverse outcomes or basic data that could facilitate the calculation of the above values.

Exclusion criteria: (a) If there was literature with identical data, studies with larger sample sizes were selected; (b) low‐quality literature (NOS score < 5 points); (c) non‐English language literature; and (d) full text could not be found.

### 2.3 Study selection and data extraction

Two researchers independently searched for and selected literature, then extracted data for cross‐checking. In the case of any differences, a third party was consulted to settle the issue. The screening process was carried out in strict accordance with the PRISMA guidance process. After reading the title and abstract of the article, literature that clearly did not meet the acceptance criteria was excluded, and articles that had initially been included were subsequently excluded after having read the full text. The basic information that was extracted included the author, publication time, country, sample size, average age, the definition of cognitive decline (assessment tools and diagnostic criteria), outcome indicators, follow‐up time, adjustment factors, and the NOS score.

### 2.4 Quality evaluation

Two researchers used the New Castle Ottawa Scale (NOS) (Stang, 2010) to independently evaluate the quality of the included literature, and a third party was consulted in the event of a disagreement. The quality evaluation table consisted of eight items, which were divided into three categories as follows: selection, comparability, and results. The total score was nine points. When the score was ≥5 points, it was deemed high‐quality literature.

### 2.5 Statistical analysis

The primary outcomes of this systematic review were considered to be all‐cause mortality and dementia. The other data were considered as secondary outcomes. The hazard ratios (HR) with 95% confidence intervals (CI) of the primary outcomes in the case of cognitive frailty compared robustly with the noncognitive frailty that were extracted from the studies for meta‐analysis.

The subgroup analyses were conducted depending upon the different assessment tools and the different models of cognitive frailty. Since the physical frailty involved both prefrailty and frailty, the cognitive frailty involved both prefrailty cognitive impairment, along with frailty and the cognitive impairment (Avila‐Funes et al., 2009; Montero‐Odasso et al., 2016; Shimada et al., 2018; Zheng et al., 2020). Ruan (Ruan et al., 2015) divided cognitive frailty into two subtypes: the reversible and the potentially reversible. The cognitive impairment of reversible cognitive frailty is SCD and/or positive biomarkers resulting from physical factors. The cognitive impairment of potentially reversible cognitive frailty is MCI (CDR = 0.5). As mentioned above, and according to the description of each research study, the types of cognitive frailty were divided into four groups as follows: cognitive impairment (CI) + frail, CI + prefrail, CI + (pre)frail, and reversible cognitive frailty.

Meta‐analysis was conducted by Stata 15.0 software. (a) Pass *I* ^2^ and the chi‐square test (the test level was *α* = 0.1) were carried out to determine the heterogeneity among the included studies. If *I* ^2^ < 50%, *p* > .1, this indicated that the heterogeneity was small, and the fixed effect model was used; if *I* ^2^ ≥ 50%, *p* ≤ .1, this indicated that the heterogeneity was large. In this case, the random effect model was used; (b) we evaluated the impact on the combined effect value after each study was eliminated individually, and a sensitivity analysis was carried out to determine whether the results were stable; (c) Beck's test and Egger's test were employed to analyze whether publication bias was present for the outcome indicators of ≥10 articles.

## 3 RESULTS

**Figure 1:**
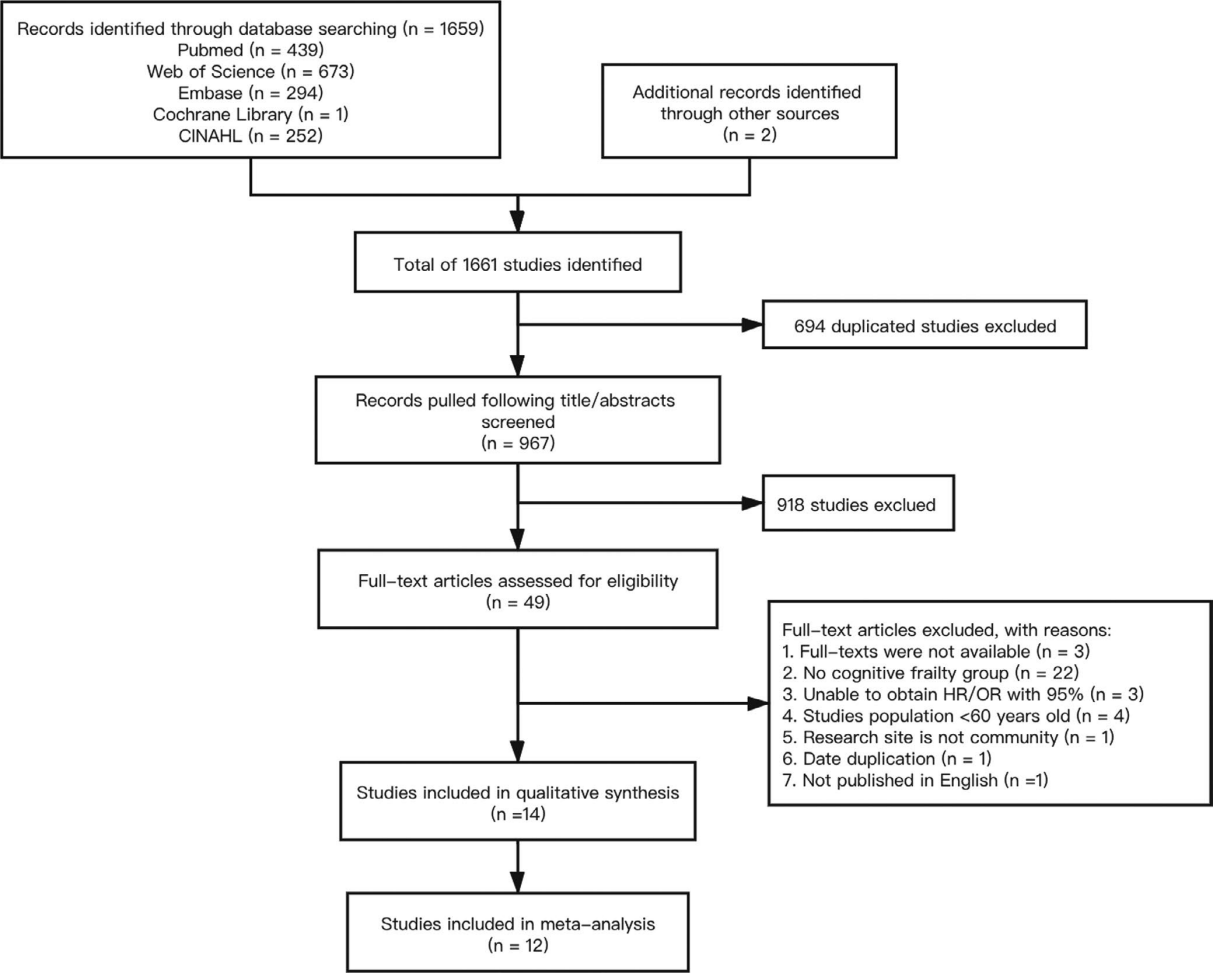
Flow diagram of the selection process of the studies. CINHAL, Cumulative Index to Nursing and Allied Health Literature; HR, hazard ratio; OR, odds ratio

A total of 1,661 documents were retrieved, of which 967 were retrieved by importing the title into the endnote software. Forty nine articles were left over after reading the title and the abstract, while 14 articles were included after reading the full text. Due to the limited number of studies on secondary outcome indicators, it was not possible to combine meta‐analyses. A descriptive analysis was carried out, and finally, 12 reports were included for meta‐analysis. The specific screening process is shown in Figure 1.

### 3.1 Characteristics of the included studies

In this study, 14 cohort studies were included. There were 57,559 subjects, and the average age ranged from 71.5 to 93.6 years old, with a prevalence of cognitive frailty between 2.5% and 50%. The subjects were followed up for a period of 2–14 years. Five assessment tools were utilized to diagnose the physical frailty, and three assessment tools were used to define the cognitive impairment. There are three studies (Avila‐Funes et al., 2009; Liu et al., 2018; Solfrizzi et al., 2017) used the Frailty Phenotype (FP) combined with the Minimum Mental State Examination (MMSE) which were the most commonly used of all the assessment tools, as illustrated in Table 1.

**Table 1:** Characteristics of included studies

Author/Year	Country	Sample	Mean age	Cognitive frailty assessment		Prevalence,%	Adverse Outcome	Effect measure	Adjusted variable	Follow‐up
				Frailty	Cognitive impairment					
Aliberti et al. (2019)	USA	7,338	74.4 ± 7.0	FP	HRS: the 27‐point scale classified as CIND (scores, 7–11)	5.0%	ADL dependence Mortality	HR	age, sex, ethnicity, education, net worth, marital status, comorbidities (stroke, hypertension, diabetes, cancer, lung disease, heart disease, and depression), and smoking status.	8 year
Avila‐Funes et al. (2009)	France	6,030	74.1 ± 5.2	FP	MMSE + IST: subjects in the lowest quartile in both tests were considered as CI	7.2%	Mobility IADL dependence ADL dependence Hospitalization Dementia Death	OR HR	age, sex, education, income, smoking status, drinking status, number of chronic diseases, self‐reported health, and CES‐D score)	4 year
Downer et al. (2019)	USA	639	82.2 ± 3.7	mFP	MMSE: <21, regardless of a participant's level of education was defined as CI	12.7%	Mortality Frailty	HR	age, sex, education, marital status, and self‐reported health conditions (diabetes, hypertension, heart disease, and arthritis)	3 year
Esteban‐Cornejo et al. (2019)	Spain	3,677	71.5 ± 7.78	FS	MMSE: ≤25 was considered as MCI	22.6%	Mortality	HR	age, sex, education, smoking, body mass index, and waist circumference	14 year
Hao et al. (2018)	China	705	93.6 ± 3.3	FI	MMSE: ≤18 was defined as CI	50%	Mortality	HR	age, sex, education levels, lifestyles	4 year
Lee et al. (2018)	South Korea	11,266	72.9 ± 6.7	mFP	MMSE‐KC: score > 1.5 *SD* below the age‐, gender‐, and education‐specific norm of MMSE‐KC was defined as CI	17.1%	Mortality	HR	age, sex, marital status, education, household income, smoking, alcohol drinking, self‐rated health, comorbidity, and depressive symptoms	3 year
Liu et al. (2018)	China	678	73.3 ± 5.3	FP	MMSE: score < 1.5 *SD* or more in each cognitive domain in the age‐ and education‐ matched norm of the same population were defined as CI	13.3%	Mortality	HR	age, sex	28 months
Montero‐Odasso et al. (2016)	Canada	252	76.6 ± 8.6	FP	MMSE + CDR: MMSE < 26 and CDR of 0.5, and without dementia	37.3%	Cognitive decline Dementia	HR	age, sex, education, number of comorbidities	5 year
Okura et al. (2019)	Japan	5,076	75.9	KCL (five items)	KCL(SR‐CD): any participant who gave a negative state answer to any of the three items was considered to have SR‐CD	13.8%	Mortality	HR	age, sex, living alone, IADL decline, isolation, oral frailty, polypharmacy, serious disease, responded method to survey, and economics.	3 year
Shimada et al. (2018)	Japan	4,570	71.9 ± 5.5	walking‐speed grip‐strength	NCGG‐FAT: one deficit of NCGG‐FAT’s domains was defined as CI	9.8%	Dementia	HR	age, sex, education level, depressive mood, and chronic medical illnesses	36 months
Solfrizzi et al. (2017)	Italy	2,150	73.2 ± 5.6	FP	MMSE + GDS: MMSE scores ≥ 15 and positive response to the item 14 of the GDS−30, and exclude dementia.	2.5%	Dementia Mortality	HR	age, sex, education, pack‐years, GDS, IADL, MMSE, Charlson comorbidity index score, and serum albumin levels	3.5/7 year
St John et al. (2017)	Canada	1751	77.5 ± 7.1	FI	MMSE: <26 was defined as CI	12.5%	Mortality	HR	age, sex, education	5 year
Tsutsumimoto et al. (2020)	Japan	9,936	73.5 ± 5.4	walking‐speed grip‐strength	NCGG‐FAT: below the standardized threshold in one or more NCGG‐FAT test were defined as CI	11.4%	Disability	HR	age, sex, education, BMI, medication, hypertension, hyperlipidemia, diabetes, stroke, osteoarthrosis, current drinking habit, current smoking habit, physical inactivity, BMI	2 year
Yu et al. (2018)	China	3,491	72.0 ± 4.9	FP	CMMSE: <21, individuals with no education, or < 24, individuals with primary education, or < 27, well‐educated individuals are identified as CI	8.7%	Poor quality of life Physical limitation Hospitalization Mortality	OR	age, sex, education, social‐economic status ladder, smoking, alcohol intake, physical activity, dietary intakes, BMI, and baseline value of the respective outcome variable	4 year 4 year 7 year 12 year

Abbreviations: CDR, Clinical Dementia Rating; CIND, cognitive impairment without dementia; CMMSE, Cantonese version of Mini‐Mental Status Examination; FI, frailty Index; FP, frailty phenotype; FS, FRAIL Scale; HR, hazard ratio; HRS, Health and Retirement Study; IST, Isaacs Set Test; KCI, Kihon Checklist; mFP, a modified version of the frailty phenotype; MMSE, Minimum Mental State Examination; MMSE‐KC, Korean version of the Mini‐Mental State Examination; NCGG‐FAT, National Center for Geriatrics and Gerontology‐Functional Assessment Tool; NOS, Newcastle‐Ottawa Scale; OR, odds ratio; *SD*, standard deviations; SR‐CD, Self‐reported‐cognitive decline.

### 3.2 Methodological quality

The 14 cohort studies were evaluated for methodological quality using the NOS. The bias risk scores of all the reports ranged between 6 and 9 (total score of 9). The included studies indicated moderate to good methodological quality and exhibited low risk of bias. The highest risk of bias in this review was assessed for Outcome and Comparability. Most of the earlier studies had not followed up long enough for the outcomes to happen (*n* = 8), whereas certain other studies did not modify in order to indicate the specific control for the second important factor (*n* = 7), as indicated in Table 2.

**Table 2:** Quality assessment of included studies based on the Newcastle‐Ottawa scale

Author/Year	Selection				Comparability	Outcome			NOS
	Representative‐ness of the exposed cohort	Selection of the non exposed cohort	Ascertain‐ment of exposure to implants	Demonstration that outcome of interest was not present at start of study	Comparability of cohorts on the basis of the design or analysis	Assessment of outcome	Was follow‐up long enough follow‐up for outcome to occur	Adequacy of follow‐up of cohorts	
Aliberti et al. (2019)	1	1	1	1	2	1	1	1	9
Avila‐Funes et al. (2009)	1	1	1	0	2	1	0	1	7
Downer et al. (2019)	1	1	1	1	1	1	0	1	7
Esteban‐Cornejo et al. (2019)	1	1	1	1	1	1	1	1	8
Hao et al. (2018)	0	1	1	1	2	1	0	1	7
Lee et al. (2018)	1	1	1	1	2	1	0	1	8
Liu et al. (2018)	1	1	1	1	1	1	0	1	7
Montero‐Odasso et al. (2016)	0	1	1	1	1	1	1	1	7
Okura et al. (2019)	1	1	0	1	1	1	0	1	6
Shimada et al. (2018)	1	1	1	1	2	1	0	1	8
Solfrizzi et al. (2017)	1	1	1	1	2	1	1	1	9
St John et al. (2017)	1	1	1	1	1	1	1	1	8
Tsutsumimoto et al. (2020)	1	1	1	1	2	1	0	1	8
Yu et al. (2018)	1	1	1	1	1	1	1	1	8

### 3.3 Primary outcomes

#### 3.3.1 All‐cause mortality

A total of 10 studies (Aliberti et al., 2019; Avila‐Funes et al., 2009; Downer et al., 2019; Esteban‐Cornejo et al., 2019; Hao et al., 2018; Lee et al., 2018; Liu et al., 2018; Okura et al., 2019; Solfrizzi et al., 2017; St John et al., 2017) described the relationship between cognitive frailty and mortality. The combined results of the included studies indicated that there was heterogeneity between the studies (*I* ^2^ = 58.3%, *p* = .004). Therefore, the meta‐analysis, which employed the random effect model, showed that the all‐cause mortality rate of older people with cognitive frailty was 1.93 times higher than that among normal healthy older people [HR = 1.93, 95% CI (1.67, 2.23), *p* < .001].

**Figure 2:**
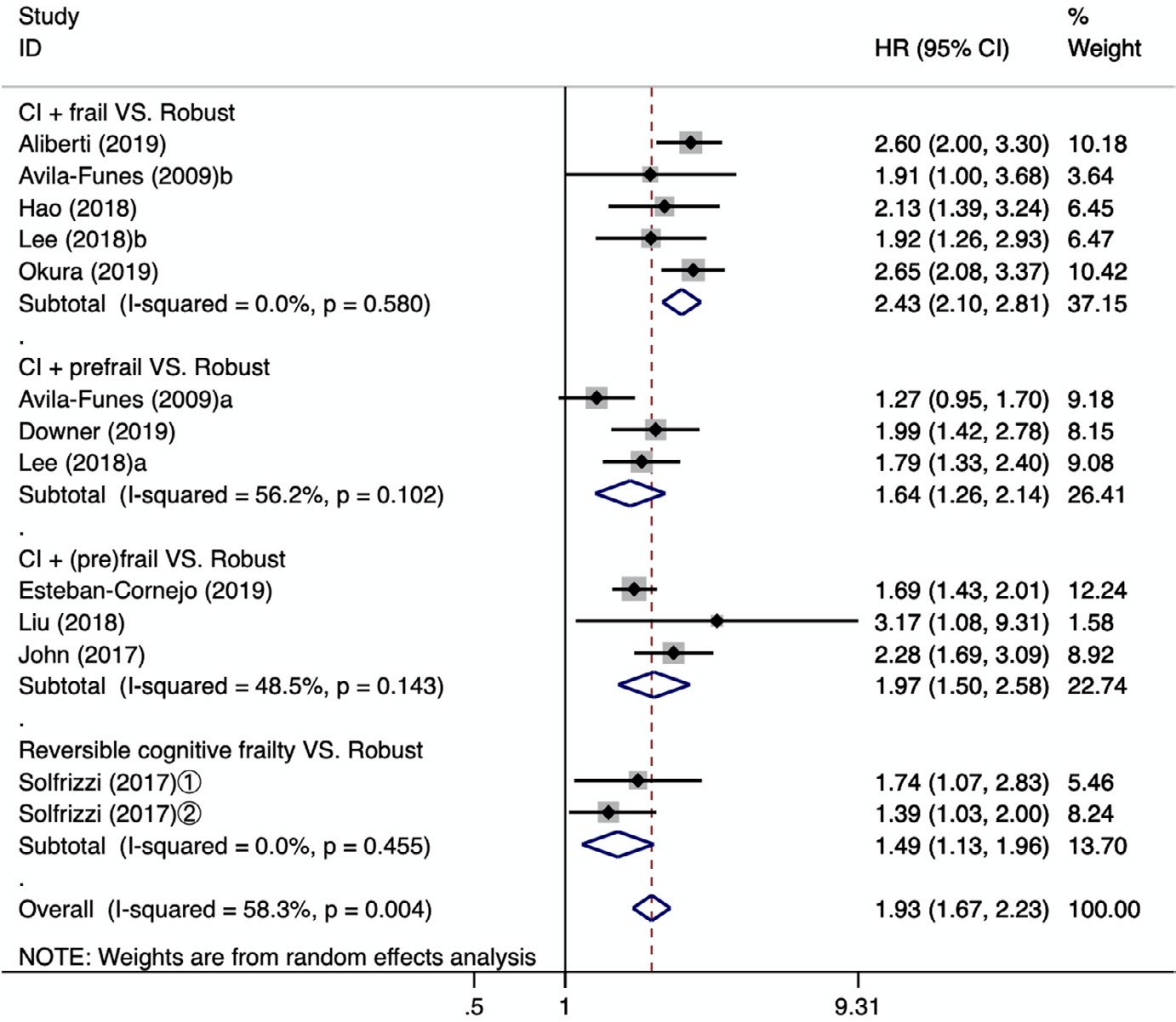
Forest plot of the association between different types of cognitive frailty and the all‐cause mortality. CI: cognitive impairment; a: cognitive impairment + prefrail; b: cognitive impairment + frail; ①: follow‐up 3.5 years; ②: follow‐up 7 years; HR: hazard ratio; 95%CI: 95% confidence interval

**Figure 3:**
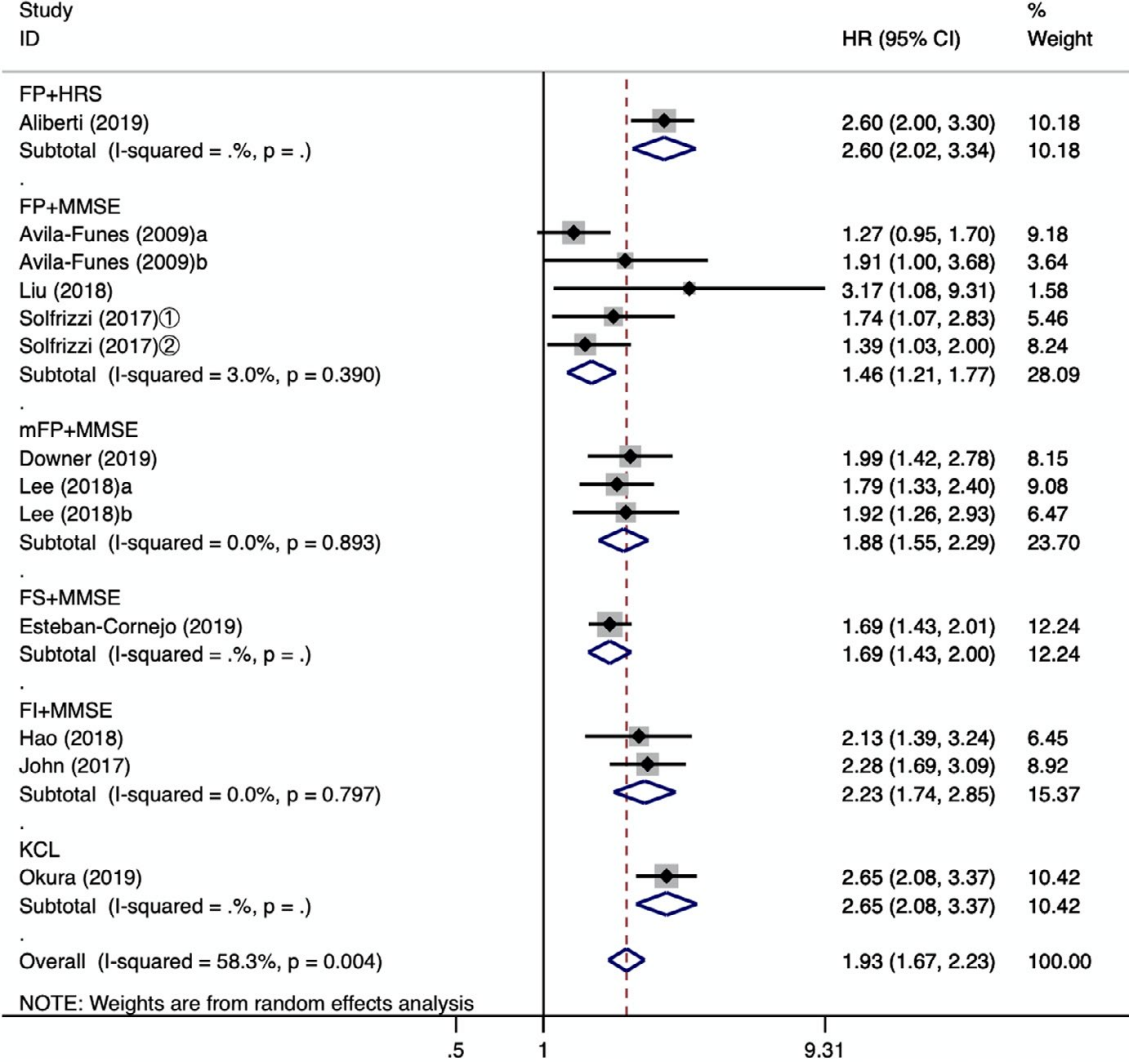
Forest plot of the association between different assessment instruments of cognitive frailty and the all‐cause mortality. FP: frailty phenotype; HRS: Health and Retirement Study; MMSE: Minimum Mental State Examination; mFP: a modified version of the frailty phenotype; FS: FRAIL Scale; FI: frailty Index; KCI: Kihon Checklist; a: cognitive impairment + prefrail; b: cognitive impairment + frail; ①: follow‐up 3.5 years; ②: follow‐up 7 years; HR: hazard ratio; 95%CI: 95% confidence interval

To explore the source of heterogeneity, this study further examined the relationship between cognitive frailty and all‐cause mortality by carrying out a subgroup analysis of models of cognitive frailty and assessment tools. The results of the subgroup analysis are shown in Figure 2. Four models of cognitive frailty increased the incidence of mortality in older people. In addition, heterogeneity among the groups decreased, and heterogeneity disappeared in the CI + frail group and in the reversible cognitive frailty group. The all‐cause mortality was the highest in the CI + frail group [HR = 2.43, 95% CI (2.10, 2.81), *p* = .001], and the combined effect was the lowest in the reversible cognitive frailty group [HR = 1.49, 95% CI (1.13, 1.96), *p* < .001]. In 10 studies, five assessment instruments were used to diagnose physical frailty and three assessment instruments were used to evaluate cognitive impairment. According to the assessment instrument, we divided them into six subgroups. After carrying out the subgroup analysis, we found that heterogeneity was significantly reduced. In addition to the two studies that could not be combined (Aliberti et al., 2019; Okura et al., 2019), the assessment method of the frailty index (FI) combined with the mini‐mental state examination (MMSE) showed that the highest risk of all‐cause mortality was in the older adults with cognitive frailty [HR = 2.23, 95% CI (1.74, 2.85), *p* = .000]. The combined effect quantity of the frailty phenotype (FP) combined with the MMSE, and the modified version of the frailty phenotype mFP) combined with the MMSE assessment tool was [HR = 1.46, 95% CI (1.21, 1.77), *p* = .000], [HR = 1.88, 95% CI (1.55, 2.20), *p* = .000], respectively, as shown in Figure 3.

#### 3.3.2 Dementia

**Figure 4:**
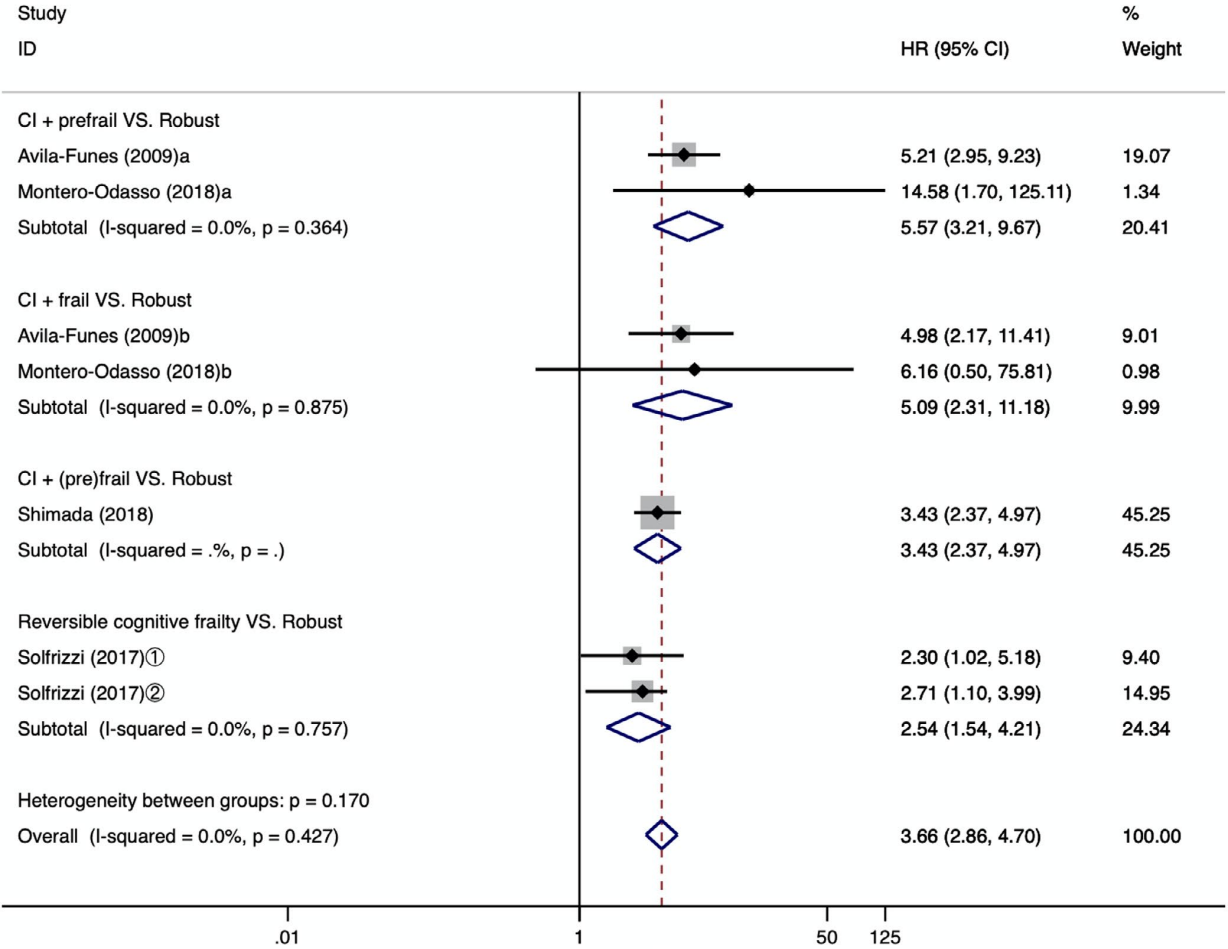
Forest plot of the association between different types of CF and dementia. CI: cognitive impairment; a: cognitive impairment + prefrail; b: cognitive impairment + frail; ①: follow‐up 3.5 years; ②: follow‐up 7 years; HR: hazard ratio; 95%CI: 95% confidence interval

Four studies (Aliberti et al., 2019; Montero‐Odasso et al., 2016; Shimada et al., 2018; Solfrizzi et al., 2017) mentioned the relationship between cognitive frailty and dementia, and there was no heterogeneity between them (*I* ^2^ = 0.0%, *p* = .427). The meta‐analysis which employed a fixed effect model showed that, compared with robust older people without cognitive frailty, the risk of dementia in older people with cognitive frailty increased significantly [HR = 3.66, 95% CI (2.86, 4.70), *p* < .001], as shown in Figure 4.

### 3.4 Other adverse outcomes

#### 3.4.1 Decreased activity

(a) Activities of Daily Living (ADLs), Instrumental Activities of Daily Living (IADLs) dependence: Two (Aliberti et al., 2019; Avila‐Funes et al., 2009) studies described the relationship between cognitive frailty and ADL, and IADL dependence. Due to the use of different statistical effects, we cannot combine the analysis. Aliberti (Aliberti et al., 2019) assessed ADL dependence according to the Katz index. After 8 years of follow‐up, it was found that people with cognitive frailty had the highest risk of ADL dependence compared with people without cognitive frailty [HR = 2.0, 95% CI (1.60, 2.60)]. Avila‐Funes (Avila‐Funes et al., 2009) used the Katz index and the Lawton Brody scale to evaluate ADL and IADL dependence. After 4 years of follow‐up, the incidence of ADL and IADLs was 9.4% and 25.5%, respectively. The results of multivariate logistic regression analysis showed that cognitive frailty was significantly related to ADL and IADL dependence [OR = 5.60, 95% CI (2.13, 14.70), *p* < .001], [OR = 3.17, 95% CI (1.47, 6.83), *p* = .003]. (b) Disability: Tsutsumumoto (Tsutsumimoto et al., 2020) used long‐term care insurance data to obtain the disability rate among the respondents, which was 5.2% during the 2‐year follow‐up period. After adjusting for confounding factors, it was found that older people with cognitive frailty had the highest risk of accidental disability compared with robust people [HR = 3.86, 95% CI (2.95, 5.05), *p* < .001], which was significantly higher than that associated with uncomplicated cognitive impairment and physical frailty. (c) Physical limitation: Yu et al. (2018) used a questionnaire to find out whether the subjects were physically restricted by difficulties in climbing stairs and moving tables and chairs. After 4 years of follow‐up, it was found that the risk of physical limitations in older people with cognitive frailty increased [OR = 1.78, 95% CI (1.26, 2.51)].

#### 3.4.2 Hospitalization

Two (Avila‐Funes et al., 2009; Yu et al., 2018) studies described the correlation between cognitive frailty and the hospitalization rate. Avila‐Funes's (Avila‐Funes et al., 2009) studies showed that the hospitalization rate of patients with cognitive frailty was 37.5% in a 4‐year follow‐up. After adjusting for confounding factors, the results showed that cognitive frailty was a risk factor for all‐cause hospitalization [OR = 1.90, 95% CI (1.09, 3.31), *p* = .02], but when the cognitive frailty had reached a stage of physical prefrailty, no correlation with all‐cause hospitalization was found, and the results were not statistically significant [OR = 0.95, 95% CI (0.68, 1.31), *p* > .05]. During the 7‐year follow‐up, Yu et al. (2018) used the cumulative length of stay to determine the relationship between cognitive frailty and all‐cause hospitalization. The results showed that compared with healthy older people, the accumulated hospitalization time of older adults with cognitive frailty increased [OR = 1.48, 95% CI (1.06, 2.06)].

#### 3.4.3 Poor quality of life

Yu et al. (2018) used the SF‐12 to evaluate the quality of life of the subjects. After 4 years of follow‐up, it was found that the risk of poor quality of life among older people with cognitive frailty at baseline was higher than that observed among robust older adults [OR = 1.53, 95% CI (1.06, 2.22)].

### 3.5 Sensitivity analysis

**Figure 5:**
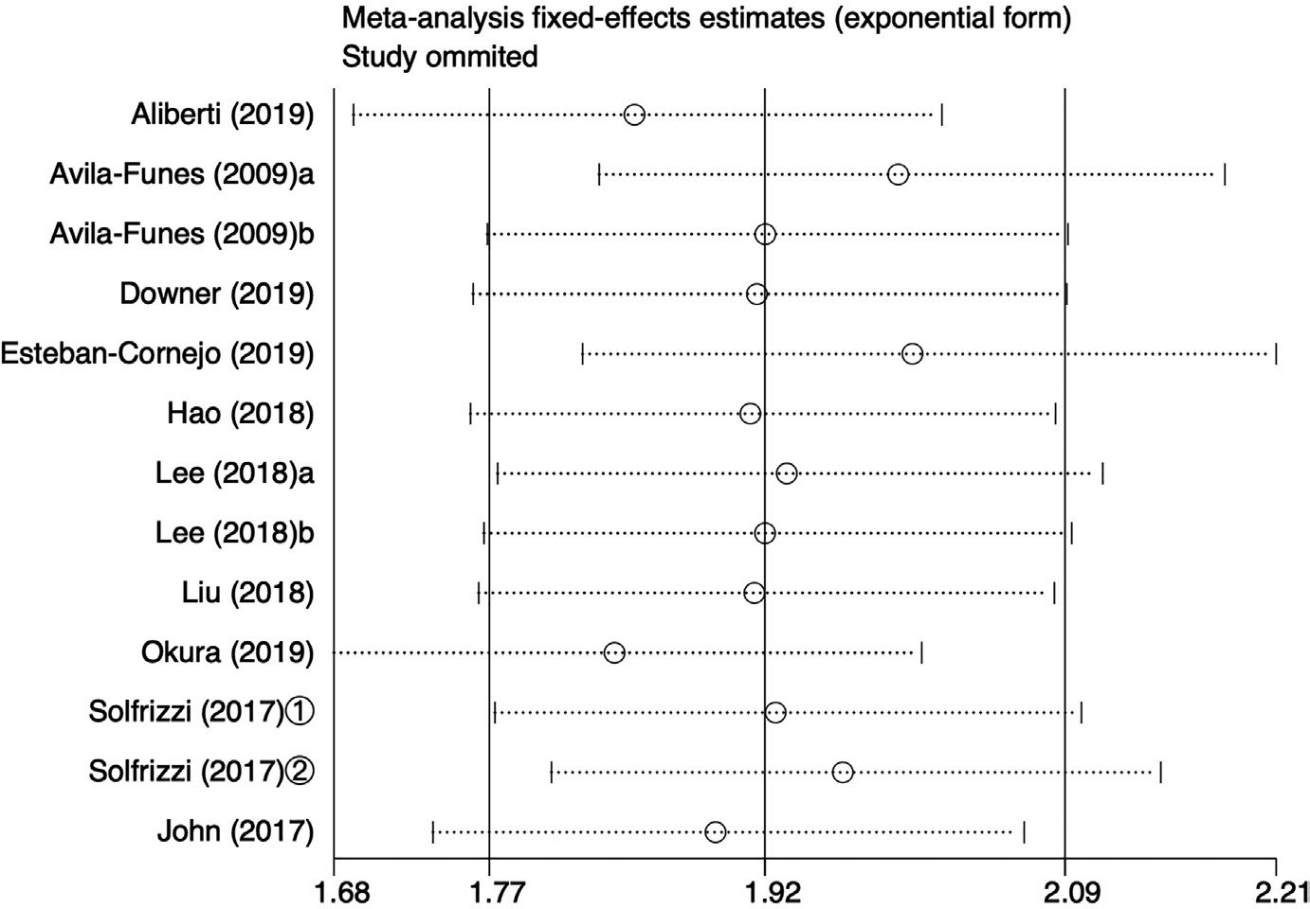
Sensitivity analysis of the effect of cognitive frailty on all‐cause mortality. a: cognitive impairment + prefrail; b: cognitive impairment + frail; ①: follow‐up 3.5 years; ②: follow‐up 7 years

A sensitivity analysis of all‐cause mortality related to the main adverse outcomes was carried out, and a small difference was found between the combined effect value and the total combined effect value after each study was eliminated, indicating that the results of this study showed high stability (see Figure 5 for details.)

### 3.6 Publication bias

**Figure 6:**
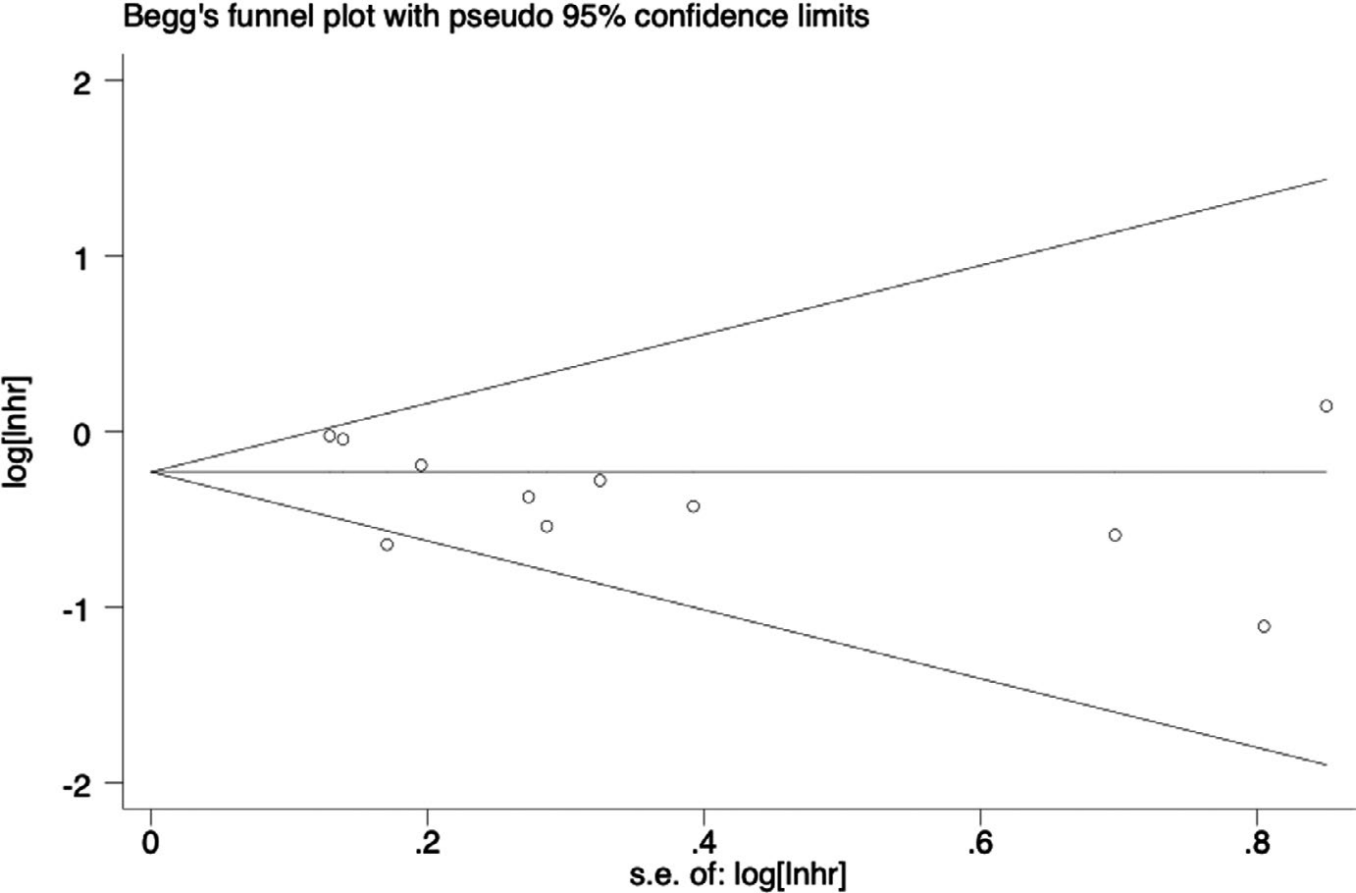
Funnel plot of the effect of cognitive frailty on all‐cause mortality. Y‐axis is expressed as loghr of HR, x‐axis is expressed as seloghr of logh

The bias analysis of all‐cause mortality was carried out using Begg's test and Egger's test. The funnel plot showed that individual studies deviated from the confidence interval, as shown in Figure 6. However, the results of Begg's test and Egger's test showed that *p* = .436 and *p* = .177, respectively, which was not statistically significant, and the possibility of publication bias was small. No published bias analysis was conducted because the number of studies on dementia included in the meta‐analysis was <10.

## 4 DISCUSSION

In this research, 14 cohort studies were used to evaluate the risk of all‐cause mortality, dementia, and other adverse outcomes of cognitive frailty, to identify the related risks and take corresponding measures to follow‐up and intervene. The results showed that the risk of all‐cause mortality, dementia, and other adverse health outcomes in older adults with cognitive frailty was higher than that in older adults without cognitive frailty. The results of the subgroup analysis, based on the types of cognitive frailty and assessment tools, did not change significantly, which was still statistically significant. It is suggested that cognitive frailty is a key risk factor for poor health outcomes among older people in the community, which has predictive value. As a heterogeneous geriatric syndrome, the pathogenesis of cognitive frailty has not been clarified, which may be related to hormones, inflammatory responses, cardiovascular risk factors, and so on (Panza et al., 2018). Cognitive impairment and physical frailty have similar time trajectories and pathological mechanisms (Furtado et al., 2019), and they interact with each other.

A meta‐analysis (Zhang et al., 2019) of the nursing homes for the frailty (alone) in older adults, indicated a high risk of mortality compared to those without frailty [HR = 1.88, 95% CI (1.57, 2.25)], while our results revealed that cognitively frailty had a higher risk of all‐cause mortality than this. Nonetheless, ZHANG's subgroup analysis displayed a higher risk of mortality [HR = 2.37, 95%CI (1.43, 5.00)] when being followed up for less than one year, whereas in another meta‐analysis in a hospital (Cunha et al., 2019), the frail (alone) individuals had a relative risk for mortality in the medium‐ [RR = 9.49, 95%CI (1.92, 46.86)] and the long‐term [RR = 7.95, 95%CI (4.88, 12.96)]. Compared to the robustness, all were significantly higher than the results of our meta‐analysis. The reason for this could have been the different study populations. Zhang and Cunha (Cunha et al., 2019) targeted the nursing home and hospital populations, respectively, with poor self‐care capabilities and more co‐morbid syndromes which raised the risk of mortality to a significant extent. Moreover, the meta‐analysis of the community‐dwelling older people indicated that frailty (alone) was significantly associated with an increased risk of developing all‐cause dementia [HR = 1.33, 95%CI (1.07, 1.67)] (Kojima et al., 2016). Our results illustrated that the community‐dwelling older adults with cognitive frailty had a higher risk of developing dementia. Hence, it could be surmised that the risk of all‐cause mortality and dementia of cognitive frailty was significantly higher than that of the physical frailty (alone), and its impact on the adverse outcomes seemed to be cumulative, with better predictive values (Especially dementia). In addition, timely intervention is also important for many negative health outcomes caused by cognitive frailty. A number of studies (Sink et al., 2015; Zhang et al., 2020) have shown that exercise can delay the progress of debilitation, prevent the occurrence of cognitive impairment, and a Mediterranean diet can also effectively prevent and improve cognitive impairment (Valls‐Pedret et al., 2015). According to the definition of cognitive frailty, we can carry out more joint interventions in many fields and modes, such as physical exercise combined with diet changes, to find effective interventions that can reduce the incidence of adverse outcomes.

At present, there is no uniform standard for the assessment of cognitive frailty, so the prevalence of cognitive decline in this study varied from 2.5% to 50%, and the results of the subgroup analysis of all‐cause mortality also showed that most of the heterogeneity derived from different assessment tools. In this study, FP is often used to evaluate physical frailty, including self‐reported fatigue, muscle strength decline, slow gait, involuntary weight loss, and a low level of physical activity. Three or more of these factors indicate frailty, while one or two suggests prefrailty (Fried et al., 2001). At present, there is no unified judgment standard for each index of FP. In terms of its application process, it needs to be adjusted in combination with the heterogeneity of people in different countries (King‐Kallimanis et al., 2014). Secondly, the results of the subgroup analysis showed that the all‐cause mortality rate of the FP combined with the cognitive impairment assessment tool group was lower than that of other groups, which may be due to the fact that the FP did not account for comorbidity, psychosocial and other conditions, which resulted in the low number of patients detected, in addition to the low positive rate of the follow‐up results. Solfrizzi (Solfrizzi et al., 2019) combined the physiological, the psychological, and the social domains to identify a new biopsychosocial frailty (BF) phenotype. The BF structure was found to be associated with the short‐ and the long‐term risks of developing overall dementia. The frailty of the older adults to dementia risk was not fully captured relatively to the comparative physiological and defect accumulation approaches, while the BF model added significant value in the case of both the assessment and the target of intervention during frailty. Some of the studies employed the frailty index (FI) (Searle et al., 2008), which is based on the cumulative health defect method to evaluate the decline, including any symptoms and signs related to adverse outcomes. The degree of decline is divided into frailty (FI > 0.25), prefrailty (FI = 0.20 ~ 0.25), and robust (FI < 0.25) (Fried et al., 2001). As the health defects in multiple dimensions are captured, the FI can facilitate more comprehensive and accurate classification criteria and risk predictions. Multiple system assessment (Kojima et al., 2018; Malmstrom et al., 2014; Theou et al., 2013) also confirms that the prediction effectiveness of FI on disability, mortality, and other adverse outcomes is better than that of other assessment instruments, which is consistent with the results of the subgroup analysis in this study. However, in practice, it is cumbersome to collect dozens of items related to health defects, which increases the workload of medical staff, and as such, the development of information technology (such as electronic medical records) can better reflect its advantages. Compared with the FI, the FP can identify debilitated patients more easily and quickly. In conclusion, it is necessary to further explore the evaluation indicators with higher sensitivity to physical frailty, and to simplify the scale and ensure its effective prediction ability, so as to avoid situations in which false positives and false negatives are reported. Ruan (Ruan et al., 2015) divided cognitive frailty into two subtypes: Reversible cognitive frailty indicates physical frailty and subjective cognitive decline, while potential reversible cognitive decline involves physical frailty and cognitive impairment. In this study, MMSE was used to diagnose cognitive impairment in cognitive frailty and to identify the criteria that were primarily adapted to study potential reversible cognitive frailty, while less attention was paid to recognizing reversible cognitive frailty. Only Solfrizzi et al. (2017) analyzed the predictive effect of reversible cognitive frailty on adverse outcomes, while subjective cognitive decline, as a precognitive impairment and form of dementia, is associated with better reversibility, which is an important stage in the secondary prevention of dementia. Therefore, in addition to the importance of reversible cognitive frailty, we should use the diagnostic criteria and screening tools of subjective cognitive decline in the early stage of mild cognitive impairment, combined with biomarkers to evaluate the cognitive decline.

Limitations of this study: (a) fewer outcome indicators that can be combined for meta‐analysis, such as the hospitalization rate, the decline in the ability to engage in activities, and other adverse outcomes; only 1–2 of such indicators, which produces incomplete results; (b) different confounding factors in every study could have certain impact on the results, while some studies did not control the additional factors; (c) there was a huge difference in the follow‐up time of the study, whereas certain studies had a follow‐up period that was too short and the adverse outcome of the study was not comprehensive; (d) unpublished gray literature was not included; and (e) due to language limitations, only English articles were retrieved.

## 5 CONCLUSION

This systematic assessment exhibited a high prevalence of the cognitive frailty in community‐dwelling older adults, while there were no uniform assessment instruments to evaluate this syndrome. This meta‐analysis had indicated that the cognitive frailty increased the risk of the all‐cause mortality and dementia among older adults. Moreover, the cognitive frailty was a better predictor of the all‐cause mortality and dementia than just frailty (alone).

## 6 IMPLICATIONS

By shedding light on the impact of cognitive frailty on the occurrence of adverse outcomes in the older adults, this study believes that the medical staff could identify the risks of cognitive frailty and the high‐risk groups as early as possible, besides taking corresponding prevention and intervention measures to reduce the occurrence of adverse outcomes. At the same time, a large‐scale, multicenter clinical investigation was carried out, and standardized assessment tools with high sensitivity and convenience were developed according to different research sites to explore the risk factors of cognitive frailty and effective intervention measures, so as to delay any further progression of the disease.

## CONFLICT OF INTEREST

No conflict of interests.

## AUTHOR CONTRIBUTION

Zihan Bu designed the study, extracted and analyzed the data, performed a meta‐analysis, and wrote the manuscript. Anle Huang performed data extraction and revised the manuscript. Mengting Xue and Qingyun Li collected the data. Yamei Bai and Guihua Xu supervised the project and revised the paper.

### Peer Review

The peer review history for this article is available at https://publons.com/publon/10.1002/brb3.1926.

## Supporting information

AppendixS1
