# Supplementary material for: Cognitive frailty as a predictor of adverse outcomes among older adults: A systematic review and meta‐analysis
Source: Brain Behav. 2020 Nov 6;11(1):e01926. doi: 10.1002/brb3.1926 (PMC7821586; doi:10.1002/brb3.1926)
Supplement: Supplementary file 1 — AppendixS1 [file BRB3-11-e01926-s001.docx]

**Appendix S1: Search strategy**

**PUBMED:**

1. **Query:** (("cognitive frailty"[Title/Abstract]) OR ("cognitive impairment"[Title/Abstract])) OR ("cognitive decline"[Title/Abstract])

**Search Details:** "cognitive frailty"[Title/Abstract] OR "cognitive impairment"[Title/Abstract] OR "cognitive decline"[Title/Abstract]

**Results:** 75,960

2. **Query:** (((frail*[Title/Abstract]) OR (pre-frail*[Title/Abstract])) OR ("frailty syndrome"[Title/Abstract])) OR ("Frailty"[Mesh])

**Search Details:** "frail*"[Title/Abstract] OR "pre frail*"[Title/Abstract] OR "frailty syndrome"[Title/Abstract] OR "Frailty"[MeSH Terms]

**Results:** 23,607

3. **Query:** "cohort stud*"[Title/Abstract] OR "prospective stud*"[Title/Abstract] OR "longitudinal stud*"[Title/Abstract] OR "Cohort Studies"[MeSH Terms]

**Search Details:** ((("cohort stud*"[Title/Abstract]) OR ("prospective stud*"[Title/Abstract])) OR ("longitudinal stud*"[Title/Abstract])) OR ("Cohort Studies"[Mesh])

**Results:** 2,190,253

4. **Query:** #1 and #2 and #3

**Search Details:** ("cognitive frailty"[Title/Abstract] OR "cognitive impairment"[Title/Abstract] OR "cognitive decline"[Title/Abstract]) AND ("frail*"[Title/Abstract] OR "pre frail*"[Title/Abstract] OR "frailty syndrome"[Title/Abstract] OR "Frailty"[MeSH Terms]) AND ("cohort stud*"[Title/Abstract] OR "prospective stud*"[Title/Abstract] OR "longitudinal stud*"[Title/Abstract] OR "Cohort Studies"[MeSH Terms])

**Filters:** from 1800/1/1 - 2020/5/10

**Results:** 439
